# Mental Health in Brazil: challenges for building care policies and monitoring determinants

**DOI:** 10.1590/S2237-96222023000200028

**Published:** 2023-04-17

**Authors:** Tânia Maria de Araújo, Mônica de Oliveira Nunes de Torrenté

**Affiliations:** 1 Universidade Estadual de Feira de Santana, Núcleo de Epidemiologia, Feira de Santana, BA, Brazil; 2 Universidade Federal da Bahia, Instituto de Saúde Coletiva, Salvador, BA, Brazil

## INTRODUCTION

A significant number of people have some type of mental disorder (MD). Global estimates indicate that worldwide, 4.4% of people suffer from depressive disorders and 3.6% from anxiety disorders,^1^ indicating that the trend for these conditions is growing steadily. In Brazil, the burden of chronic noncommunicable diseases (NCDs) has increased substantially,^2^ ^),(^ ^3^ to the point that mental disorders account for one third of all NCD cases.^4^ ^)-(^ ^6^ Brazil ranks fourth among Latin American countries with the highest annual increase in suicides; in absolute numbers, it is in second place in this region of the Americas in absolute numbers, it is in second place in this region of the Americas.^7^ MDs are relevant, due to the direct adverse effects they produce and the impacts they generate on the quality of life and health of affected populations. There is evidence that mental illness is associated with an increase in the frequency and severity of other chronic diseases,^8^ increased absenteeism at work^9^ and excessive disabilities.^3^ ^)^ This evidence highlights the need to pay attention to these conditions in Brazil. 

The MDs become relevant in the overall health situation of the Brazilian population, important barriers to addressing them can be identified, in particular (i) social/regional inequalities in access to treatment, (ii) absence, insufficiency or inadequacy of care policies and (iii) invisibility of the contexts and/or conditions that produce this illness. Noteworthy among these obstacles is the backsliding suffered by the mental health care model, systematically implemented by the psychiatric counter-reform in recent years, implying significant setbacks in the adoption of inclusive models of psychosocial care. 

With regard to inequalities, indicators of mental health service availability, for example, draw attention to the magnitude of difficulties in accessing services and, in this sense, also to the marked differences between Brazil’s macro-regions. The 2013 National Health Survey (Pesquisa Nacional de Saúde - PNS)^10^ showed that 78.8% of Brazilians with moderate or severe depressive symptoms did not receive any type of treatment for this condition, with significant regional discrepancies: in the Northern region, the proportion of untreated individuals was 90.2%, while in the Southern region it was 67.5%.^10^

Mental disorders, therefore, represent a major challenge for the Brazilian National Health System (Sistema Único de Saúde - SUS), and for the prevention/promotion of mental health and care, specialized assistance and rehabilitation networks in the country. This article sheds light on some points of the debate, which are crucial for the process of building a mental health policy guided by the principles of the right to human dignity and social inclusion. 

## CHALLENGES TO BUILDING PUBLIC POLICIES ON MENTAL HEALTH CARE

The relevance of the mental health policy in Brazil is recognized nationally^11^ and internationally,^12^ resulting from the Brazilian Psychiatric Reform (Reforma Psiquiátrica Brasileira - RPB), the text of which was approved and sanctioned by the National Congress in 2001. It is a community-based model of psychosocial care, converging with the premises of an anti-asylum, deinstitutionalizing and wide-ranging care model. Despite this recognition, important gaps remain in this policy, especially with regard to its implementation, which is conditioned to regional factors and, currently, to reduced budget allocation despite the population’s growing mental health demands, affected by social inequalities that deepen the degree of illness and suffering, generating lack of care.^13^

The original RBP proposal was to replace the biomedical care model, focused on single-purpose psychiatric hospitals and excessive use of psychotropic drugs, with a model developed based on practices centered on subjects, territories and human rights, as advocated by the World Health Organization (WHO).^14^ As such, the RPB was responsible for rescuing thousands of people suffering from mental illness, who until then had been segregated, helping them to return home and share spaces for social inclusion, moving around within their cities, exercising citizenship as protagonists.^15^

Regrettably, the process of implementing and developing this model has suffered a huge setback in recent years, reflecting changes in legislation and psychiatric counter-reform policies.^16^ ^),(^ ^17^ This setback has led to the reintroduction of psychiatric hospitals within the psychosocial care network, in 

addition to significant financial support given to therapeutic communities (TCs), responsible for the confinement of thousands of people, including children and adolescents, especially those who abuse alcohol and other drugs. In 2017, the Instituto de Pesquisa Econômica Aplicada (IPEA)/Ministério da Economia mapped 1,986 TCs in Brazil and identified 74.3% of them as being installed in rural areas, 73.2% with dormitories for four to six people and 30.1 % with availability for 70 to 300 people.^18^ The predominant profile of these services is their location in isolated areas and the considerable number of individuals per dormitory. Alongside the investment in TCs, funding for open health services and housing spaces for people with mental suffering has been reduced; furthermore, access to social protection and income programs has been made difficult, such as the Disability Benefit (Benefício de Prestação Continuada - BPC) and the Return Home Program (Programa de Volta para Casa - PVC), in addition to income generation and solidarity economy programs.

The setbacks to the RPB, plus the increase in MDs in Brazil and worldwide, including among health workers and workers in precarious situations, and the health and humanitarian crisis triggered by the COVID-19 pandemic,^19^ have led to the worsening of situations of vulnerability and mental health problems, increased needs for care and demands for new strategies for addressing the situation.^20^

Given this context, the challenges imposed on the (re)building of mental health care policies in Brazil are immense. They involve not only care models but also, and above all, the need for social transformations. Building inclusive models involves overcoming social structures inherited from the past, anchored in structural racism and patriarchy. These structures, fueled by exploitation processes based on financial and neoliberal capital, have not only kept these dynamics intact, but have also exacerbated the situation, based on a series of recent reforms that have deepened social inequalities, accentuated income concentration and which, consequently, have led to the exclusion of significant portions of the population. Psychosocial care centered on social inclusion and on the production of autonomy for populations suffering from mental illness implies overcoming these structures, which generate oppression and subordination. There is, therefore, a multisectoral and multidimensional task to be carried out, as to defining a care and assistance model and, on a larger scale, a dispute between different models of social production and reproduction.

In the current moment of reaffirmation of the Brazilian democratic State, the possibility of furthering the RPB model and making radical changes to it emerges, repositioning the fight for an anti-asylum, anti-racist, anti-patriarchal, anti-prohibitionist, decolonial, socially fair and emancipatory psychiatric reform.^21^ Turning this utopia into reality depends, on the one hand, on agility in implementing the mental health policy based on RPB principles - some of which are listed in Box 1 -, and on the other hand, on the active participation of relevant stakeholders - the academy and the social movements -, especially those that bring together mental health service users and their families, human rights organizations, educators and legal system workers, in the re-establishment of the fundamental premises for the guidance of mental health policies at the federal, state and municipal levels. An important initial step towards resuming the psychosocial care model will be to ensure the repeal of a set of 25 ordinances, laws, resolutions and decrees, as called for in the document entitled “Repeals needed for a Policy on Mental Health, Alcohol and other drugs” (*Revogações necessárias para uma Política de Saúde Mental, álcool e outras drogas*), drawn up by a significant group of social movements, including Rede Nacional Internúcleos da Luta Antimanicomial (Renila), Frente Ampla em Defesa da Saúde Mental (FASM), Movimento de Usuários, Associação Brasileira de Saúde Mental (Abrasme), Associação Brasileira de Saúde Coletiva (Abrasco) and Conselho Nacional de Saúde (CNS). The resumption of a wide-reaching debate, foreseen at the 5th National Conference on Mental Health, on the theme “Mental Health Policy as a Right: for the defense of care in freedom, towards progress and guarantee of SUS psychosocial care service” (“A Política de Saúde Mental como Direito: pela defesa do cuidado em liberdade, rumo a avanços e garantia dos serviços da atenção psicossocial no SUS”), is another initiative that will bring fundamental inputs to guide mental health policies in Brazil. 

**Box 1:** Guiding principles of the mental health policy for the Psychosocial Care Network (Rede de Atenção Psicossocial - RAPS)

Principles for the RAPS	Guidelines
1. Mental health care	Must be universal, equitable and integral.
2. Forms of service organization	Must be robust, broad, territorialized, accessible, qualified and integral in order to replace psychiatric hospitals.
3. Diversity	Must be culturally sensitive and socially adequate to accommodate the social, plural and diverse mental health needs of different regions, populations (Indigenous, Black, Gypsy, etc.) and social segments (women, children, elderly, adolescents, LGBTQIA+ people etc.).
4. Inclusive human rights-based care	Must be deinstitutionalizing, with actions aimed at people’s social reintegration and the guarantee of their human rights and dignity.
5. Qualified and comprehensive care (for crises)	RAPS workers must deal with psychosocial crises quickly and efficiently, by networking and at all levels of care. For this, adequate training, continuing education and the necessary resources must be guaranteed.
6. Dynamic and complex conceptions	Among health workers/managers, conceptions of mental suffering must be encouraged as processes of dynamic and synergistic intersection of eco-ethnobiosociopsychological conditions. Workers must be provided with adequate training to work at these levels of complexity.
7. Expanded intervention	Health strategies must be built on non-medical, non-iatrogenic and non-pathologizing bases.
8. Drug policy	The drug policy must be built from an anti-prohibitionist perspective, with harm reduction as a guideline for the care offered.
9. Measuring the size of the problem and its determinants	Analysis/diagnosis of the mental health situation must guide actions and strategies. With the help of epidemiological and social science methods, the main mental health problems that affect different social groups/segments (according to race/skin color, gender, social class, age, territory, among others) must be identified and analyzed, in order to build strategies to address these problems, taking into account the intersectional nature of how they are produced.
10. Funding	The Federal Government budget allocation for the mental health policy must be close to 5% of the total health budget, as recommended by the World Health Organization.

## CONCLUSION

This brief article has systematized points for discussing challenges to mental health policies in Brazil, based on the principles of defending rights and human dignity, anchored in collective processes of building alternatives and innovation. These points do not exhaust the list of demands, needs and challenges, rather their purpose is to foster a debate that, we hope, will be fruitful. 

The current Brazilian political scenario, characterized by the strengthening of democratic principles of social organization, reaffirmation of the SUS and the creation of a Department of Mental Health within the SUS, points to the emergence of a rich process of discussion and definition of a more effective mental health care model. We are therefore facing a unique opportunity for aspects related to psychosocial care to be highlighted in the discussion about the Brazilian National Health System and mental health in Brazil. As stated above, the challenges are many, pointing to the development of a community network of mental health services that meets territorial demands, capable of providing adequate and universal care, supported by investments in monitoring the conditions that produce suffering and mental illness, its determining and conditioning factors. The long trajectory of the struggles and the experiences accumulated in this direction, as well as the successes achieved, show the ability to overcome and produce innovative initiatives. It is fitting returning to the original path intended to be taken by the RPB, also in the form of a tribute to the many people who are dedicated to the work and defense of the RPB, strengthened by the demand of thousands of Brazilian citizens whose dignity needs to be recovered and assured.
